# Violence at work in emergency care units: nurses’ experiences*

**DOI:** 10.1590/1518-8345.3856.3323

**Published:** 2020-08-12

**Authors:** Camila de Souza Oliveira, Júlia Trevisan Martins, Maria José Quina Galdino, Renata Ribeiro Perfeito

**Affiliations:** 1 Universidade Estadual de Londrina, Londrina, PR, Brazil.; 2 Universidade Estadual do Norte do Paraná, Bandeirantes, PR, Brazil.

**Keywords:** Worlplace Violence, Nurses, Occupacional Health, Emergency Medical Services, Aggression, Public Health, Violência no Trabalho, Enfermeiras e Enfermeiros, Saúde do Trabalhador, Serviços Médicos de Emergência, Agressão, Saúde Pública, Violencia Laboral, Enfermeras y Enfermeros, Salud Laboral, Servicios Médicos de Urgencia, Agresión, Salud Pública

## Abstract

**Objectives:**

to understand the perception of nurses in emergency care units about the violence experienced at work.

**Method:**

qualitative study conducted through 21 individual interviews between November and December 2018 in two emergency care units in a city in Paraná. Symbolic Interactionism was adopted as the theoretical framework and the Thematic Content Analysis technique was used to evaluate the data.

**Results:**

from the thematic category experiencing psychological violence in the nurses’ daily work, it was evidenced that it was related to threats against their lives, cursing, humiliation, embarrassment, attempt to defame them, as well as pressure from subordinates. In the category experiencing physical violence in the nurses’ daily work, it was found that it was imposed through pushing, pulling hair, throwing objects, the presence of firearms and knives and, even, witnessing murder.

**Conclusion:**

nurses suffered acts of violence by external and internal people, from the emergency care units themselves. Managers, nurses and society need to look reflexively and critically at the violence that happens and implement actions to avoid them, thus providing a safe working environment for all involved and educate society in order to make the reduction of violence a priority in public policies.

## Abstract (pt)

**Objetivos:**

compreender a percepção de enfermeiros de unidades de pronto atendimento sobre a violência vivenciada no trabalho.

**Método:**

estudo qualitativo realizado por meio de 21 entrevistas individuais entre novembro e dezembro de 2018 em duas unidades de pronto atendimento de uma cidade paranaense. Foi adotado o Interacionismo Simbólico como referencial teórico e a técnica de Análise de Conteúdo Temática para avaliar os dados.

**Resultados:**

da categoria temática vivenciando a violência psicológica no cotidiano laboral do enfermeiro evidenciou-se que esteve relacionada com ameaças contra as suas vidas, xingamentos, humilhações, constrangimentos, tentativa de denegri-los, bem como pela pressão dos subordinados. Já na categoria vivenciando a violência física no cotidiano laboral do enfermeiro verificou-se que foi impetrada por meio de empurrões, puxões de cabelo, arremesso de objetos, presença de armas de fogo e de facas e, até, o presenciamento de assassinato.

**Conclusão:**

os enfermeiros sofreram atos de violência por pessoas externas e internas, das próprias unidades de pronto atendimento. Os gestores, os enfermeiros e a sociedade precisam olhar reflexiva e criticamente para as violências que acontecem e implementar ações para evitá-las e, assim, propiciar um ambiente laboral seguro para todos os envolvidos, sensibilizando a sociedade para que a redução da violência seja uma prioridade nas políticas públicas.

## Abstract (es)

**Objetivos:**

comprender la percepción de los enfermeros de unidades de atención de emergencia sobre la violencia experimentada en el trabajo.

**Método:**

estudio cualitativo realizado a través de 21 entrevistas individuales entre noviembre y diciembre de 2018 en dos unidades de atención de emergencia en una ciudad de Paraná. El Interaccionismo Simbólico se adoptó como marco teórico y la técnica de análisis de contenido temático se utilizó para evaluar los datos.

**Resultados:**

desde la categoría temática situaciones de violencia psicológica en la práctica cotidiana de los enfermeros, se evidenció que la misma se hallaba vinculada con amenazas contra sus vidas, insultos, humillaciones, situaciones embarazosas, intento de denigración, así como la presión de los subordinados. En la categoría situaciones de violencia física en la práctica cotidiana de los enfermeros, se descubrió que se imponía a través de empujones, tirones de cabello, arrojando objetos, con la presencia de armas de fuego y cuchillos e, incluso, presenciando asesinato.

**Conclusión:**

los enfermeros sufrieron actos de violencia por parte de personas externas e internas, a las propias unidades de atención de emergencia. Los gerentes, las enfermeras y la sociedad deben mirar reflexiva y críticamente la violencia que ocurre e implementar acciones para evitarla y, de ese modo, ofrecer un ambiente de trabajo seguro para todos los involucrados, además de crear conciencia en la sociedad para que la reducción de la violencia se convierta en prioridad de las políticas públicas.

## Introduction

Defining violence at work is complex and, therefore, there is no consensus on its concept; in the present study is understood as negative behavior or action in a relationship between two or more people, marked by aggressiveness, which can occur repeatedly or unexpectedly, including situations in which workers are intimidated, threatened, assaulted or subject to offensive acts in work-related circumstances^(^ 1 ^)^.

Violence may occur mainly psychologically, verbally and physically, which can lead the professional to illnesses, psychosocial problems and decreased interest in work. Violence can be caused by both external and internal aggressors, that is, when the aggressors are the institution’s own workers^(^ 2 ^)^.

Psychological violence is the intentional use of power against a person or community; aims to control actions, behaviors, beliefs and decisions, resulting in problems for the person’s physical, mental, spiritual, moral or social development. Its subdivisions are verbal aggression, moral harassment, sexual and racial discrimination^(^ 3 ^)^.

Verbal violence is understood as the transgression of verbal rules, which humiliates, degrades, disrespects the dignity and worth of the person^(^ 1 ^)^. Moral harassment is understood as humiliating behavior, which disqualifies or demoralizes; it is repeated and aims to demote a worker or group of workers during their work^(^ 4 ^)^.

Violence in the work environment has been increasing significantly in recent times in all professional areas but, when it comes to health professionals, this environment becomes even more prone to this kind of occurrence^(^ 5 ^)^. A study carried out by the Regional Nursing Council of São Paulo (*Conselho Regional de Enfermagem de São Paulo*, COREn-SP in Portuguese language) found that 75% of nurses have already suffered some kind of violence in their work environment^(^ 6 ^)^.

It is emphasized that violence is present in any area of nursing practice, but there is a predominance in emergency rooms and emergency units, places that have a greater flow of patients, adverse work conditions, as well as, there is underreporting of these occurrences creating an environment, in which violent acts became susceptible to acceptance, making their prevention and combat difficult^(^ 7 ^)^.

A study revealed the negative impacts on physical, social, and especially psychological health among nursing workers^(^ 8 ^)^. Research carried out in Madrid showed that workers when exposed to violence have higher rates of anxiety, distress and burnout syndrome than those who have not suffered any type of violence^(^ 9 ^)^.

Due to the impacts on the psychic health of people who suffered violence at work, this study sought support in the theory of Symbolic Interactionism, since it is based on three premises: the first reveals that the relationship that people have with the world is based on the meaning it presents; the second, indicates that these meanings are the result of the interaction with other people and the third explains the modification of these meanings, according to the process that the person goes through^(^ 10 ^)^.

Thus, in Symbolic Interactionism, each gesture performed by people corresponds to a symbol that gives rise to the different meanings and objects that surround people^(^ 10 ^)^. Therefore, it is believed that this theory is of fundamental importance to analyze behaviors, since each symbol generates an individual behavior and has a unique meaning for each person, which applies to the nurses’ interpretation of the perception of violence, since, different situations generate different symbols and meanings according to the interaction that each professional had with the object of the study, that is, with violence.

Given the above considerations and the impact on workers’ health, this study aims to answer the following question: what is the perception of nurses in emergency care units about workplace violence? To this end, the objective was to understand the perception of nurses in emergency care units about the violence experienced at work.

It is believed that this study may alert nursing professionals and consequently, other professionals of the area to recognize the problem, as well as to encourage other workers to reflect on the subject, being able to identify situations potentially capable of causing acts of violence or even to perceive the physical, verbal and psychological aggressions, that are still considered intrinsic to the health work environment. It is assumed that it can also collaborate with managers, indicating how and where to invest, in order to provide a safer and risk-free environment, increasing the quality of life at work and, thus, preventing health problems and promoting the workers’ well-being.

## Method

Descriptive study with a qualitative approach, carried out in the only two public emergency care units (*Unidades de Pronto Atendimento*, UPA) in a city in the north of the State of Paraná, which serve approximately 400 patients, each one, *per* day and with a 24-hour daily operation.

By intentionality, the 24 nurses who make up the staff of the two UPA were invited, meeting the following inclusion criteria: being a member of the active staff effectively, working in both shifts (day - from 7 a.m. to 7 p.m. and night - from 7 p.m. to 7 a.m.) and having suffered some kind of violence in the workplace. Those who were on vacation and/or licenses of any kind were excluded. Thus, to define the number of participants, all were approached; however, one refused to participate and two were on leave, totaling 21 nurses.

Data were collected during the period from November to December 2018, through semi-structured individual interviews, with an average duration of 31 minutes, recorded and conducted by the first author of the study, based on the following guiding question: how do you perceive workplace violence in your work? Participants were previously contacted by phone, after a pilot test, according to the number informed by the coordinators of each UPA and the interview was scheduled in person according to the availability informed by the professional. In order to guarantee privacy and the minimum of discomfort, the interviews were conducted in a private and reserved place at the participant’s own work institution.

For data collection, the literature recommendation^(^ 11 ^)^ was followed, the interviews were carried out until saturation occurred, that is, the convergence/repetition of the statements, which happened to 16 nurses; however as the intentionality technique was used and all possible participants were invited to compose the study previously, it was decided to interview the total sample, that is, the 21 nurses. The recorded interviews were transcribed shortly after their performance by two people and checked by a third person, in order to maintain scientific rigor. Transcripts will be archived for five years and recorded statements have been deleted.

For the analysis of the collected data, the Content Analysis technique was used in the thematic modality, which occurred in three moments: 1) pre-analysis: in which the initial ideas were systematized and the indicators for the interpretation of the collected information were identified, following the principles of completeness, representativeness, homogeneity and relevance; 2) exploration of the material, in which the coding and identification of the registration and context units took place and 3) treatment, inference and interpretation of the results^(^ 11 ^)^.

Still, to deepen the interpretation of the data, the theory of Symbolic Interactionism was used, which understands the way individuals interpret objects and people, symbolizes the circumstances and analyzes the processes of socialization, behaviors and change of opinions, being flexible according to the situations defined by them^(^ 12 ^)^.

This theory proposes that the human being attributes different meanings to the objects around and according to his/her relation with the universe, together with the expression of psychological factors, this meaning is generated through human interaction and the same object can have different meanings for people, as each individual attributes different factors as responsible for the change in the value it assumes. Thus, Symbolic Interactionism assimilates that life and the beings present in it are constantly modified, according to the changes that occur in the universe of the respective objects^(^ 10 ^)^.

The study was guided by the principles of Resolution of the National Health Council 466/2012 and 510/2016, which regulate research involving human beings. The participants signed the Free and Informed Consent Form (ICF) and the research was carried out after approval by the Research Ethics Committee, opinion nº 2,732,477. To preserve the anonymity of the respondents, the statements are presented using the letter E for nurse (enfermeiro in Portuguese language), followed by the numbering from 1 to 21.

## Results

Of the 21 participants in the study, the average age was 42 years old, the youngest being 32 and the oldest 51 years old. Only four of them were men. They all had been working at the UPA for about three years and worked 30 hours a week. Eleven worked during the day, eight at night and two of them replaced colleagues’ shifts, having no fixed schedule. The average training time was 16 years and the income was between R$3,200.00 (três mil e 200 reais in Portuguese language) and R$9,000.00 (nove mil reais in Portuguese language), which corresponds to US$825 and US$2,320 dollars (dollar value on June 15^th^, 2019: R$3,88 - tres reais e oitenta e oito centavos in Portuguese language).

After the analysis and interpretation of the collected data, two thematic categories emerged: Experiencing psychological violence in the nurse’s daily work and Experiencing physical violence in the nurse’s daily work.

Experiencing psychological violence in the nurses’ daily work

In their statements, the nurses revealed that psychological violence happens routinely when they are developing their activities. They feel emotionally pressured and intimidated by patients who constantly threaten to expose them in the media, complain to the ombudsman and even make threats against their lives. *Some even draw a firearm to intimidate us. We experience great psychological pressure (E9); Now everything is about filming and then, patients start recording everything. Employees also make pressure, everything is overtime, they always want more and more overtime (E10); Patients threaten like this: I know who you are, I know what your car is and I know that you are the head nurse; they show the pen knife, knife and even a firearm in their waist. They raise their blouses or lower their pants so that we can see that they are armed and understand the message. I already assisted patients with a pen knife, a knife and even a firearm in their waist (E12); I was here one day and he came to coerce me, he was screaming in my face and filming me and the population follows this idea (E14); Sometimes we catch patients intimidating us with knives, they show the knives under their blouses and threaten us. On the internet it is written like this: we have to cut off their heads, we have to make minced meat from them (E15); They say: we are going to get you, I’ll wait for you outside, it won’t be like this. I had to make a Police Report because I was threatened by a boy who said he was going to end my life and that of my colleagues too (E16); The patient said: don’t you know where I live? You don’t know who I am, I can get you out there, see? (E17).*

Still, in relation to psychological violence, nurses verbalized that they experience it, through screams and curses, perpetrated by patients and companion and, also, attribute some causes for this violence, as well as the measures taken. *When the waiting time is longer, patients say: you are not doing anything and I am here waiting, sometimes we need to make a Police Report at the police station (E1); There was a patient who screamed at me and the doctor, he kept screaming and we ended up going to the police station to make a Police Report (E3); Violence defames you as a professional and as a human being. The patients say I pay your salary and you don’t work (E6); They have already told me like this: you treat me well because I’m a drug dealer and they start threatening. They speak badly, despise and say that the public servant is a tramp and that the public servants are all the same (E7); The companions curse us and scream, mainly when it takes too long for them to be assisted (E8).*

Respondents also stated that they suffer psychological violence that is imputed by co-workers. *Depending on how you correct the employee, there is a threat that you will be prosecuted for moral harassment. It is very complicated to be a boss, we have the responsibility for the service, but the harassment of employees is huge (E4); They verbalize it like this: my coworker is wrong and you only see failure in me and then internal communication of moral harassment comes ( E6).*

Experiencing physical violence in nurses’ daily work

In their testimonies, the nurses revealed that they suffered physical violence and witnessed it in their workplaces and also revealed some factors that contribute to this violence. *A patient almost broke the door because he thought we were taking too long to assist him, but the demand is great for few human resources (E1); There was a murder, an execution here at the UPA, they went in and shot the patient in the head in front of everyone, it was two or three headshots. I don’t even like to remember. We also deal with psychiatric patients, aggressive and violent, there was one who took scissors, but we managed to hold his hand. The other took the needle and threatened whoever came to hold him. There was a Police Report, everything is registered. There was a try to rescue a bandit who was being treated. A psychiatric patient broke the arm of an employee who tried to defend a colleague. We made a Police Report (E2); A patient dropped everything that was on the counter, the police was close and started a persecution and there was death on the street below the UPA. There was also a fugitive who came in here and shot all over the place and killed the person inside the UPA (E5); The patient came to me, grabbed my hair, pulled and dropped me on the floor (E7); There was a case of people throwing bricks at us and breaking the computer (E9); These days, the colleague from the administration was punched in the head there at the service desk (E14); A woman threw a metal water jug at us and a receptionist was beaten with a punch in the face and the attacker had a knife in the waist (E15); I have already witnessed the reception staff suffering kicks and punches several times (E17); The husband beats the wife and then comes to the UPA to get satisfaction because he thinks we are meddling in the woman’s life, he even opened the door to the doctor’s office and almost managed to attack him (E20).*

## Discussion

Unveiled through Symbolic Interactionism, the meanings of objects and social products are pointed out according to the interaction that the subject has with the object and the meaning that he/she has attributed to it^(^ 10 ^)^. In the case of workplace violence suffered and reported by nurses, each situation has a meaning and a consequence according to the experiences and the social interaction that the victim shows with other people. This meaning can only be attributed to those who have suffered violence according to their own perceptions.

Studies have shown that psychological violence has been more common than physical violence among nursing professionals^(^ 13 ^-^ 14 ^)^ with an average of 2.29 episodes of verbal aggression per eight-hour shift versus 1.18 of physical aggressions^(^ 15 ^)^.

Psychological violence has become practically routine in the work environment of nurses working in public emergency sectors^(^ 16 ^-^ 17 ^)^. It is noteworthy that the frequency of insults and other types of psychological violence is often considered normal, making this situation almost natural in everyday work. Thus, reflections and coping strategies are necessary so that this trivialization does not become common place in health institutions^(^ 14 ^,^ 18 ^)^.

In this study, the statements show that abuse is practiced by patients and companions who have an exalted behavior and are nervous about the delay in attending. It is a fact that the difficulty in accessing health care, as well as the delay in care, collaborate for these people to behave aggressively and use psychological violence against health professionals, in order to try to guarantee their rights^(^ 19 ^)^. Other studies have also revealed that the acts of violence perpetrated against nurses have as their main causes the delay in attendance, in addition to the reduction in staff and resources and the waiting time was identified as the main triggering factor for insults in the emergency sector^(^ 20 ^-^ 22 ^)^.

The nursing team reports suffering workplace violence due to the frustrations of patients with the health service, which, in most cases, are related to the lack of human resources and insufficient materials to meet the demand, combined with the lack of work organization, as sources that facilitate acts of violence. Still, patients and companions can become violent when they perceive the poor quality of services or the lack of professional commitments or, also, when they realize that their rights are being neglected^(^ 18 ^)^.

It is noteworthy that the excessive demand of patients in the emergency service can result in a decrease in the quality of care and, in turn, provoke feelings of anxiety, frustration and loss of control, which may translate as a possible factor for the violent behavior from patients^(^ 7 ^)^.

In Symbolic Interactionism, society is conceived as a communication network and, consequently, defined in a different way that gives social reality its complex, dynamic and in continuous symbolic transformation character^(^ 23 ^)^. It is believed that such assumptions can be referred to the values that are present in the culture of violence against nurses, in which the professional as well as the patients are often the only ones to blame for this situation, when it is known that violence has multiple casualties, such as: poverty, decreased social chances, political interests, economic interests, drug use, among others^(^ 24 ^)^.

In this study, the statements showed that the nurse feels the psychological pressure coming from his/her subordinates. These data are analogous to those of Iranian research, which pointed out that professionals feel humiliated by coworkers’ insults in front of patients^(^ 25 ^)^. Daily exposure to this kind of violence results in negative impacts that affect the mental health of nurses in emergency units^(^ 7 ^)^.

In the interviewees’ statements, the fundamentals of Symbolic Interactionism can be perceived, in which nurses acted based on the sense that things have for them. This sense can be manipulated and modified through a constant interpretive process, used by these professionals to deal with situations arising, especially from their subordinates^(^ 26 ^)^.

With regard to verbal violence, the intention to humiliate or cause fear was clearly identified in the statements of the interviewees in this study. Chilean research has identified that this type of violence is the main suffered among workers of a public emergency service, with the patient and family members responsible for most of the abuses; however, a minority of professionals reported/recorded the incident and none filed legal action against the aggressor^(^ 27 ^)^. This fact differs from the statements of the nurses in this investigation, since, as attitudes against violence, they issued Police Report registered in police stations.

Studies show that it is necessary to guarantee the safety of health workers in order to prevent injuries, leave and abandonment of employment, as well as social isolation and the intention to quit work^(^ 14 ^,^ 28 ^)^.

It should be noted that nursing professionals should be aware of the violence suffered in the workplace and report it when they are victims in any situation, since there are repercussions for mental, social and quality of life at work for those who suffer violence, whatever the type^(^ 18 ^)^.

It is necessary for nurses to have attitudes to recognize and report violence in all applicable instances, so that this problem can have greater visibility and, thus, enable government spheres, nursing councils, their unions and health institution managers to plan and program measures to prevent violence and protect nurses^(^ 29 ^)^. It is known that daily exposure to insult, disrespect, humiliation or any kind of violence coming from exalted companions, patients and/or co-workers cause damage to workers’ mental and physical health^(^ 30 ^)^.

An American study showed that 54% of nurses who were questioned about violence, said that they feel safe in the work environment, due to a zero tolerance policy, while 16% believe that nothing is enough to allow them to feel safe in these environment^(^ 31 ^)^.

With regard to physical violence, in the present study, the nurses’ reports were related to kicks, punches, fractures of limbs, throwing objects and even shooting with firearms. These data are similar to those of an Australian study that identified that spits, bites and attempts to assault workers’ homes were also acts of violence perpetrated against nurses in the emergency department^(^ 21 ^)^.

Furthermore, it is known that emergency services, such as UPA, are characterized as being at high risk for acts of violence, since they are frequently attending patients with complex health status and those using alcohol and drugs, which make them more aggressive, and consequently, their companions also become aggressive, collaborating to the increase of aggressions^(^ 17 ^,^ 27 ^,^ 32 ^)^. One in ten nursing professionals has already suffered some type of physical aggression and, when suffering it, the probability of being away from work increases, in addition to the psychological consequences that he/she will have to deal alone, due to lack of support from managers^(^ 32 ^)^.

Regarding the limits of the study, it is indicated that the data presented reflect the reality of nurses from two UPA in only one city. However, because there are few national studies on workplace violence with nurses from these institutions, it is believed that it contributes to the advancement of scientific knowledge, by revealing information that may facilitate the development of action strategies, with the purpose of increasing the safety of these workers and consequently, promote well-being in the workplace. It should also be noted that in the qualitative research it is not intended to generalize the results, since the data are subjective in nature, unveiled at a given moment in people’s lives, they are unique to the situation that is being experienced. It is noted that the results will be presented and discussed with all respondents, including managers.

## Conclusion

Nurses suffered acts of psychological violence in the verbal form of an external and internal nature, coming from the emergency care units themselves and physical violence performed by people external to the work environment.

It is necessary that managers together with nurses look, reflexively and critically, at the phenomenon of violence that happens to UPA nurses and implement actions to avoid or minimize them and, thus, provide a safe working environment for the ones involved. It is also essential to sensitize the society, the unions and the bodies responsible for public health, so that occupational violence is a priority in policies, in class bodies and also the target of studies by the scientific community.
